# The Potential Use of DNA Methylation Biomarkers to Identify Risk and Progression of Type 2 Diabetes

**DOI:** 10.3389/fendo.2015.00043

**Published:** 2015-03-30

**Authors:** Linn Gillberg, Charlotte Ling

**Affiliations:** ^1^Diabetes and Metabolism, Department of Endocrinology, Rigshospitalet, Copenhagen, Denmark; ^2^Faculty of Health and Medical Sciences, University of Copenhagen, Copenhagen, Denmark; ^3^Epigenetics and Diabetes Unit, Department of Clinical Sciences, Lund University Diabetes Centre, Malmö, Sweden

**Keywords:** biomarkers, DNA methylation, epigenetics, prediction, prevention, type 2 diabetes

## Abstract

Type 2 diabetes mellitus (T2D) is a slowly progressive disease that can be postponed or even avoided through lifestyle changes. Recent data demonstrate highly significant correlations between DNA methylation and the most important risk factors of T2D, including age and body mass index, in blood and human tissues relevant to insulin resistance and T2D. Also, T2D patients and individuals with increased risk of the disease display differential DNA methylation profiles and plasticity compared to controls. Accordingly, the novel clues to DNA methylation fingerprints in blood and tissues with deteriorated metabolic capacity indicate that blood-borne epigenetic biomarkers of T2D progression might become a reality. This Review will address the most recent associations between DNA methylation and diabetes-related traits in human tissues and blood. The overall focus is on the potential of future epigenome-wide studies, carried out across tissues and populations with correlations to pre-diabetes and T2D risk factors, to build up a library of epigenetic markers of risk and early progression of T2D. These markers may, tentatively in combination with other predictors of T2D development, increase the possibility of individual-based lifestyle prevention of T2D and associated metabolic diseases.

The complex multi-organ disease type 2 diabetes mellitus (T2D) is difficult to predict and cure. Abnormalities in glucose metabolism and states of intermediate hyperglycemia are established long before overt T2D develops (1, 2). Therefore, prediction of pre-diabetes and early T2D progression enables individuals at high risk to reduce the likelihood of developing the disease through lifestyle changes (3–5). Epigenetics, which can be described as heritable, cell-specific modifications of the DNA and its associated proteins, which can alter the expression of genes, has been implicated in the pathogenesis of T2D and other complex age-related diseases (6–15). Methylation of cytosine residues in DNA (DNA methylation) is the most studied epigenetic trait, and recent data show significant influences of age and lifestyle related risk factors such as overweight and physical activity on site-specific DNA methylation in blood and tissues relevant for T2D (11, 14, 16–20). This review will discuss the potential of identifying DNA methylation biomarkers to predict development of T2D.

## The Heterogeneity of Type 2 Diabetes

The main risk factors for T2D are age, overweight defined by a high body mass index (BMI), an unhealthy diet, reduced physical activity, an adverse intrauterine environment, and an unfavorable genetic predisposition (4, 5, 21–23). Large-scale studies indicate that the incidence of T2D and pre-diabetes can be reduced through lifestyle changes such as non-smoking, physical activity, improved diet, and weight loss (3, 24). Studies suggest insulin resistance in the liver and peripheral tissues (skeletal muscle and adipose tissue) as a predictive marker of future T2D development (2, 25, 26). Insulin resistance in different tissues together challenges the pancreatic beta cells to produce and secrete increased amounts of insulin. The capacity of insulin production at these challenged states largely determines the likelihood of T2D development. Although several of the identified genetic risk variants of T2D seem to influence insulin processing and secretion (27), the individual capacity of beta cell function during long-term challenged states cannot yet be predicted. Similarly, there is a lack of predictive markers of the individual capability to maintain glucose homeostasis during challenged metabolic conditions such as overweight, which is determined by key metabolic features such as hepatic and peripheral insulin sensitivity, hepatic glucose production, as well as secretion and action of hormones derived from the intestines, adipocytes, and the brain. To optimize the preventive care of T2D, prediction of the disease should be achieved at an early stage. This could potentially be achieved through epigenetic biomarkers that, in combination with measurements of blood metabolites and possibly also identification of genetic variants associated with T2D, may link the heterogenic etiology and pathogenesis of T2D.

## Age Associated DNA Methylations in Blood and Diabetes-Related Tissues

In 2005, a study by Fraga et al. revealed that the pattern of DNA methylations and histone modifications in several tissues were more diverse in monozygotic twin pairs who were older, had different lifestyles, and had spent less of their lives together (28). This study was among the first to demonstrate that environmental factors and age can have lifelong impacts on the phenotype by altering DNA methylations across multiple human tissues.

The number of studies that demonstrate highly significant correlations between age and site-specific DNA methylation in diverse human populations is rapidly increasing. Convincing data obtained with both site-specific and epigenome-wide approaches has contributed to the identification of specific cytosines in the human genome where either the addition or removal of an electrophilic methyl group is significantly associated with age (11, 18, 19, 29–37). These studies have advanced the research of human aging, suggesting that epigenetics is involved in the age-related gradual decline of cellular functions that, opposed to chronological age, is referred to as biological age (38). Indeed, two independent but highly overlapping predictive models of aging, characterized by the DNA methylation levels of 71 and 353 genomic CpG sites respectively, have been developed from epigenomic data derived from several human tissues and cell types obtained with Illumina Infinium HumanMethylation450 BeadChips (34, 39). These “epigenetic age clocks” may be promising markers of human aging (34, 39, 40). Knowledge of the individual aging process could increase our understanding of why some individuals develop complex, age related diseases such as Alzheimer’s, cancer, cardiovascular disease, and T2D.

Many of the DNA methylations which represent strong age-related associations in blood are situated in CpG islands of genes important for human metabolism, which are not functional in blood cells but are highly expressed in metabolically active tissues such as adipose tissue, liver, heart, and skeletal muscle (31, 32, 35, 36, 41, 42). Interestingly, CpG methylation of the krüppel-like factor 14 gene (*KLF14*), which has been genetically linked to T2D and to high-density lipoprotein cholesterol levels in genome-wide association studies (GWAS) (43, 44), was identified in two of the studies mentioned above (34, 39). Thus, if age-associated DNA methylations in blood reflect DNA methylations in other tissues, these epigenetic modifications might be involved in age-related diseases and pathologies.

Indeed, an increasing amount of studies demonstrate that distinct age-associated DNA methylations in blood are present in other human tissues including skeletal muscle (45, 46), kidney (45), and the brain (33, 45). Together, these results point toward a general “epigenetic aging clock” across several human tissues (34), and furthermore indicate that age-induced DNA methylations may affect gene transcription and function. Hereby, they may serve as possible indicators of age-associated progression of insulin resistance and T2D.

## DNA Methylations Associated with Lifestyle-Related Traits in Blood and Diabetes-Related Tissues

Recently, Dick et al. reported significant associations between BMI and site-specific DNA methylation of the gene encoding hypoxia inducible factor 3 alpha (*HIF3A*) in blood from 479 individuals, as well as in adipose tissue from another population of 635 women (16). This study demonstrates that specific DNA methylations in blood that are associated with a lifestyle-related trait can reflect DNA methylations in other tissues. To date, few other studies report significant associations between DNA methylation and BMI or fat distribution in human blood or adipose tissue (47–50), and more studies are needed to consolidate these findings and to discover novel descriptive and mechanistic clues to the association between adipose tissue DNA methylation and overweight. In addition, epigenetic markers of pancreatic beta cell function, glucose tolerance, and insulin sensitivity in liver and peripheral tissues are required in the search for epigenetic biomarkers of T2D development.

The DNA methylation levels and plasticity of CpG sites in the promoter region of the metabolic regulator *PPARGC1A* have been extensively studied in relation to T2D. *PPARGC1A* encodes PGC1α, which is a transcriptional co-activator that regulates expression of numerous genes with a key role in mitochondrial function (51). Hitherto, *PPARGC1A* promoter methylation represents the best example of site-specific DNA methylation alterations in insulin secretory and insulin responsive tissues from T2D patients (12, 52, 53) and individuals at increased risk of T2D (53–56). Significant correlations between *PPARGC1A* promoter methylation and insulin sensitivity have been reported in skeletal muscle (57) and liver (58). Moreover, blood DNA methylation at four loci in *PPARGC1A* predicted adiposity in children up to 14 years (59). Accordingly, besides age and BMI, associations between DNA methylation and key features responsible for hyperglycemia, such as insulin resistance and beta cell dysfunction, could potentially be present in T2D-relevant tissues. If mirrored in blood, these DNA methylations may constitute predictive markers of T2D progression.

## Epigenetic and Non-Epigenetic Biomarkers for Diabetes Progression

The research field of epigenetic biomarkers for metabolic diseases is still in its infancy. Among the studies reporting potential epigenetic markers of T2D with predictive or diagnostic character, Hidalgo et al. identified a CpG site in a gene important for cholesterol transport (*ABCG1*) with DNA methylation levels that were significantly associated with fasting insulin and HOMA-IR in CD4^+^ T cells from 837 non-diabetic individuals (60). Another study examined epigenome-wide methylations in blood from twins discordant for T2D, that were followed up with replication and omics analyses, and identified DNA methylation alterations in *MALT1* (which has a role in the nuclear factor-κB pathway) as well as the G-protein receptor 6 gene (*GPR61*), that were suggested to reflect T2D progression (61). In addition, effective screening of the blood-borne human epigenome in relation to T2D was conducted by Toperoff et al. who identified DNA hypomethylation of specific sites in young individuals who later developed T2D (62). Also adipose tissue specific CpG sites in numerous genes associated with T2D (*PPARG, IRS1*, and *TCF7L2*) were shown to exhibit differential DNA methylation in individuals with T2D compared to healthy controls (13). *PPARG* encodes a transcription factor with a key role in adipose tissue and *IRS1* encodes insulin receptor substrate one which is involved in insulin signaling. Additionally, *TCF7L2* encodes a transcription factor involved in the Wnt signaling pathway. However, these markers of T2D or pre-diabetes need to be further replicated in future studies.

Small non-coding RNAs (e.g., miRNA and lncRNA) are yet another possible source of biomarkers for disease progression (63, 64). Recently, Guay et al. reviewed the potential of using the microRNA profile in blood as an estimates of health status and identified differential miRNA profiles in patients with type 1 diabetes (T1D) and T2D as well as distinct miRNAs which appear to be predictive of long-term complications of diabetes (65). Furthermore, another study reported associations between risk of diabetic nephropathy in T1D cases and blood methylation at 19 CpG sites (66).

Importantly, human experiments aiming to identify diverse markers of T2D progression based on genetic (family history, GWAS SNPs), epigenetic (DNA methylation, histone modifications), transcriptomic, metabolomic, and anthropometric measures, and the combination of these, are ongoing (14, 67–70).

## Clinical Utility of Future Epigenetic Biomarkers

The current enthusiasm for identification and clinical application of epigenetic biomarkers for early pathological states in complex non-communicable diseases is great. Whereas many DNA methylation biomarkers of T2D are awaiting discovery, a diagnostic test for the early detection of colorectal cancer through blood-borne DNA hypermethylation of the *SEPT9* promoter is available and used in the clinic today (71). In short, the Septin9 test is simply a detection of DNA methylation levels in the v2 region of the *SEPT9* promoter in blood plasma which is sampled in the clinic and analyzed in the laboratory (71–73). Here, hypermethylation of the *SEPT9* promoter is identified in cell-free DNA that has been released into the bloodstream from tumor cells. In the field of T2D, early identification of more subtle epigenetic markers that mirror tissues with deteriorated metabolic function is desirable. Nevertheless, the strong evidence that an increased risk of complex metabolic diseases originate in early life (21, 74, 75) opens up possibilities of discovering DNA methylation biomarkers that may help to estimate the individual susceptibility of future T2D development (76). To this end, it is noteworthy that we in a recent study identified reduced DNA methylation and increased gene expression of *SEPT9* in pancreatic islets of T2D individuals compared with non-diabetic controls, supporting the role of this gene also in T2D (11).

## Perspectives and Future Challenges

Novel research in the field of epigenetics opens up new opportunities of identifying biomarkers for risk and progression of complex metabolic diseases such as T2D. Through epigenome-wide as well as site specific DNA methylation characterization, new information about tissue-specific and tissue-general associations between DNA methylation and age or lifestyle related risk factors of T2D are rapidly revealed. Further insight to these associations will ideally improve the variety and quality of existing predictive T2D biomarkers, and thereby increase the possibilities to postpone, or prevent, T2D in individuals at high risk for the disease.

Epigenetic research will also increase our current understanding of epigenetic patterns and plasticity in humans. Interestingly, mechanistic studies of DNA methylation regulation, recently reviewed by Gabriella Ficz (77), reveal tight associations between cellular signaling pathways and DNA methylation. This implies that external, environmental stimuli are involved in the regulation of epigenetic modifications. Besides further understanding of DNA methylation regulation in different human tissues via external signals and age, mechanistic and descriptive epigenetic studies will increase our understanding of how DNA methylation is inherited and determined by genetic variants (14, 29, 77–79). Recent reports of DNA methylation changes induced by lifestyle-related factors such as exercise and overfeeding (14, 20, 80) together with the appearing impact of genetic variation on DNA methylation levels (18, 78, 79) in metabolically active tissues, suggest that specific DNA methylations might be phenotypic mediators of both environmental and genetic effects. Further descriptive as well as mechanistic studies are required to reveal how these hypothetical genome–epigenome–environment interactions might influence human health and disease, and perhaps also help us to find the “missing heritability” of T2D (10, 79). In support of functional and disease-related interactions between genetics and tissue-specific epigenetic patterns, the NIH Roadmap Epigenomics Mapping Consortium published some of the most recent human epigenomic data, where they demonstrate epigenomic enrichments in tissues related to a trait which had previously been associated with a genetic variant (81). An additional layer to this complicated network is the memory of fetal development. Since the epigenetic fingerprints were established during fetal life, an adverse intrauterine environment may be memorized as differences in DNA methylation patterns and plasticity in adult individuals and thereby increase their risk of T2D (23, 54, 55, 82–85).

Future challenges include targeted research that will bring us closer to a better understanding of epigenetic features such as the environmentally influenced plasticity of DNA methylation in different tissues, the influence of heritability and genetic variation on DNA methylations, the epigenetic influence on transcriptional regulation, as well as the mechanistic control of specific DNA methylations that are associated with either age- or lifestyle-related phenotypes.

Prevention of T2D through lifestyle changes can be improved through early discovery of T2D progression. Future research on epigenetics and T2D will possibly result in an archive of epigenetic markers that might aid in the individually targeted prevention of T2D and associated metabolic diseases.

## Author Contributions

LG and CL wrote the work and approve its publication.

## Conflict of Interest Statement

The authors declare that the research was conducted in the absence of any commercial or financial relationships that could be construed as a potential conflict of interest.
